# Supplementary material for: Complexome profiling of the Chlamydomonas psb28 mutant reveals TEF5 as an early PSII assembly factor
Source: Plant Cell. 2025 Mar 17;37(6):koaf055. doi: 10.1093/plcell/koaf055 (PMC12164590; doi:10.1093/plcell/koaf055)
Supplement: koaf055_Supplementary_Data [file koaf055_supplementary_data.zip › psb28_interactive.zip!/plots/intensity/qualityPlots/peptides/_Cre13.g602650.t1.2.html]

### Cre13.g602650.t1.2

Trivial name:   
SeqCoverage [median]: 23.20  
Mass: 29.11 kDa  
  
 

| Sequence | MissedCleavage | Mass [Da] | wtIntensity | muIntensity | Score |
| --- | --- | --- | --- | --- | --- |
| ADEVPTVGDVDIR | 0 | 1384.68 | 10991.70 | 8339.94 | 127.42 |
| GLGSVDGALK | 0 | 915.50 | 3333.28 | 1809.64 | 111.17 |
| NVTGSDIK | 0 | 832.43 | 36471.39 | 11023.51 | 148.99 |
| VRNVTGSDIK | 1 | 1087.60 | 8694.18 | 0.00 | 78.14 |
| WDAQVVEGK | 0 | 1030.51 | 20440.77 | 3711.96 | 148.52 |
| YAGIGGLSEAIGWTEVQQEQNK | 0 | 2377.16 | 524.04 | 0.00 | 74.50 |
|  |  |  | 80455.4 | 24885.0 |  |
